# Inductively powered wireless pacing via a miniature pacemaker and remote stimulation control system

**DOI:** 10.1038/s41598-017-06493-5

**Published:** 2017-07-21

**Authors:** Parinaz Abiri, Ahmad Abiri, René R. Sevag Packard, Yichen Ding, Alireza Yousefi, Jianguo Ma, Malcolm Bersohn, Kim-Lien Nguyen, Dejan Markovic, Shervin Moloudi, Tzung K. Hsiai

**Affiliations:** 1 Department of Medicine, David Geffen School of Medicine at UCLA, Los Angeles, CA 90095 USA; 2 Department of Bioengineering, School of Engineering and Applied Sciences, UCLA, Los Angeles, CA 90095 USA; 3 Department of Electrical Engineering, School of Engineering and Applied Sciences, UCLA, Los Angeles, CA 90095 USA; 4 VA Greater Los Angeles Healthcare System, 11301 Wilshire Blvd, Los Angeles, CA 90073 USA; 5 Present Address: Beihang University, Beihang, China

## Abstract

Pacemakers have existed for decades as a means to restore cardiac electrical rhythms. However, lead-related complications have remained a clinical challenge. While market-released leadless devices have addressed some of the issues, their pacer-integrated batteries cause new health risks and functional limitations. Inductive power transfer enables wireless powering of bioelectronic devices; however, Specific Absorption Rate and size limitations reduce power efficiency for biomedical applications. We designed a remote-controlled system in which power requirements were significantly reduced via intermittent power transfer to control stimulation intervals. In parallel, the cardiac component was miniaturized to facilitate intravascular deployment into the anterior cardiac vein. Given size constraints, efficiency was optimal via a circular receiver coil wrapped into a half-cylinder with a meandering tail. The pacemaker was epicardially tested in a euthanized pig at 60 beats per minute, 2 V amplitude, and 1 ms pulse width, restoring mean arterial pressure from 0 to 37 mmHg. Power consumption was 1 mW at a range of > 3 cm with no misalignment and at 2 cm with 45° displacement misalignment, 45° x-axis angular misalignment, or 45° y-axis angular misalignment. Thus, we demonstrated a remote-controlled miniaturized pacing system with low power consumption, thereby providing a basis for the next generation of wireless implantable devices.

## Introduction

Despite great advancements in implantable cardiac pacemaker technology, complications from pacemaker leads continue to compromise nearly 10% of all implants^1, 2^. These complications may include lead dislodgement, extracardiac stimulation, vascular occlusion and hemorrhaging, and electrical abnormalities from insulation breaks and conductor coil fracture^3, 4^. Lead implants additionally increase the risk for infection to the heart valves and erosion of the conduction fibers^5^. Other complications include tissue puncture during the implant procedure, causing perforation and rupture of the heart or air entrapment in the lungs known as pneumothorax^6^. Once implanted, the pacemaker lead-tissue interface develops fibrosis and adhesion to the cardiac chamber, rendering lead removal a life-threatening procedure^7^. For these reasons, we sought to develop a wireless pacing system which can eliminate many of the risks associated with lead-based devices.

These motivations have also generated a trend for pacemaker manufacturers towards the development of leadless devices. Most prominent are the Micra and Nanostim^8^. Although the recent FDA approval of the Micra marks a positive step towards leadless devices, it introduces a new set of complications, notably due to their integration of a battery into the main body of the pacer^8–10^. Alternative solutions for leadless devices have been proposed, including utilization of energy harvesters^11, 12^ and ultrasound-based wireless pacers^8, 13, 14^. However, these are similarly hindered by a large device size that leads to mechanically stressed fixation mechanisms. Extensive work has been done on wireless power transfer via an inductive link, including nearfield, midfield, and farfield transmission mechanisms^15–21^. However, inductive power transfer introduces numerous bioengineering challenges due to Specific Absorption Rate (SAR) limits and minimal size capacity for device implantation.

Power efficiency of an inductive link depends on two primary factors: (1) the quality factor of the transmitting and receiving antennas, and (2) the coupling coefficient. Both present significant design challenges. The quality factor, *Q*, represents the ability of the resonant circuit to retain energy and is heavily influenced by transmission frequency, *f*, as shown in Eq. (1)^22^:

1 $$Q=\frac{2\pi fL}{R},$$

where *L* is antenna inductance and *R* is the effective ohmic losses.

While a higher frequency increases the quality factor, it also leads to an increase in tissue absorption. This presents two key challenges: (1) the device must adhere to guidelines established by the Federal Communications Committee (FCC) for radio frequency (RF) SAR limits, and (2) increase in tissue absorption leads to decreased power transfer from the transmitter antenna to the receiver antenna^23^. Moreover, increasing frequency results in decreased efficiency of rectification, thus presenting an additional limitation on the parameter selection.

The coupling coefficient, *k*, is heavily influenced by antenna geometry, as shown in Eq. (2)^24^:

2 $$k=\frac{{d}_{1}^{2}{d}_{2}^{2}}{\sqrt{{d}_{1}{d}_{2}}{(\sqrt{{d}_{1}^{2}+{D}^{2}})}^{3}},$$

where *d*_1_ is transmitter antenna diameter, *d*_2_ is receiver antenna diameter, and *D* is distance between antennas.

Antenna geometry also impacts the inductance, which in turn influences the quality factor, as shown in Eq. (1). However, the dimensions of the device are restricted to the small size capacity of the cardiac implant location. The dimensions of the receiving unit, which will be in contact with the cardiac tissue, must be maintained below a few millimeters to prevent mechanical stresses on the fixation anchor, thus ultimately leading to significant reductions in the coupling coefficient.

Together, the quality factor and coupling coefficient determine power transfer efficiency as shown in Eq. (3)^25^:

3 $${\rm{\eta }}=\frac{{k}^{2}{Q}_{1}{Q}_{2L}}{1+{k}^{2}{Q}_{1}{Q}_{2L}}.\frac{{Q}_{2L}}{{Q}_{L}},$$

where *Q*_1_ is the quality factor of the transmitter antenna, *Q*_2_ is the quality factor of the receiver antenna, ${Q}_{2L}={Q}_{2}{Q}_{L}/{Q}_{2}+{Q}_{L}$, and ${Q}_{L}={R}_{Load}/2\pi f{L}_{2}$.

Previous work on inductively powered medical devices has often focused on improving power transfer efficiency. However, given the anatomical and physiological constraints for medical implants, there is a limited capacity for adjusting the variables that control the amount of power delivery in an inductively powered wireless system. To address both the size and absorption requirements, we sought to instead direct our design criteria towards the system architecture itself, specifically by minimizing the power-consuming functionalities and components of the wireless power receiving unit. In this context, we aimed to develop a remote-controlled miniaturized pacing system with significantly reduced power consumption. Notably, despite the pacer size reduction to allow deployment inside the anterior cardiac vein, the subcutaneously positioned transmitting unit in the thorax was able to provide continuous pacing at 60 beats per minute (BPM), 2 V voltage amplitude, and 1 ms pulse width. We established a low power rating of less than 1 mW at a wireless range of > 3 cm with no misalignment, at 2 cm with 45° displacement misalignment, at 2 cm with 45° x-axis angular misalignment, and at 2 cm with 45° y-axis angular misalignment. This significant power reduction was made possible through the concept of remote stimulation, whereby the control system is located within the transmitter to eliminate the need for a power harvesting unit inside the wireless power receiving unit. Accordingly, our design introduces the possibility for a secure vascular fixation strategy while providing sufficient power transfer for our leadless implantable pacemaker.

## System Design

A pacing system was developed with two key design criteria: (1) to resolve the mechanical failure points in the fixation mechanism by extensive miniaturization of the pacer, and (2) to achieve sufficient wireless power transmission from the power source to the geometrically constrained pacer. The former would allow for implantation external to the cardiac chambers to avoid high intra-cardiac pressure gradients, while enabling intravascular deployment of the device to the anterior cardiac vein. The latter would be achieved without exceeding SAR limits.

### Control system design

**Figure 1:**
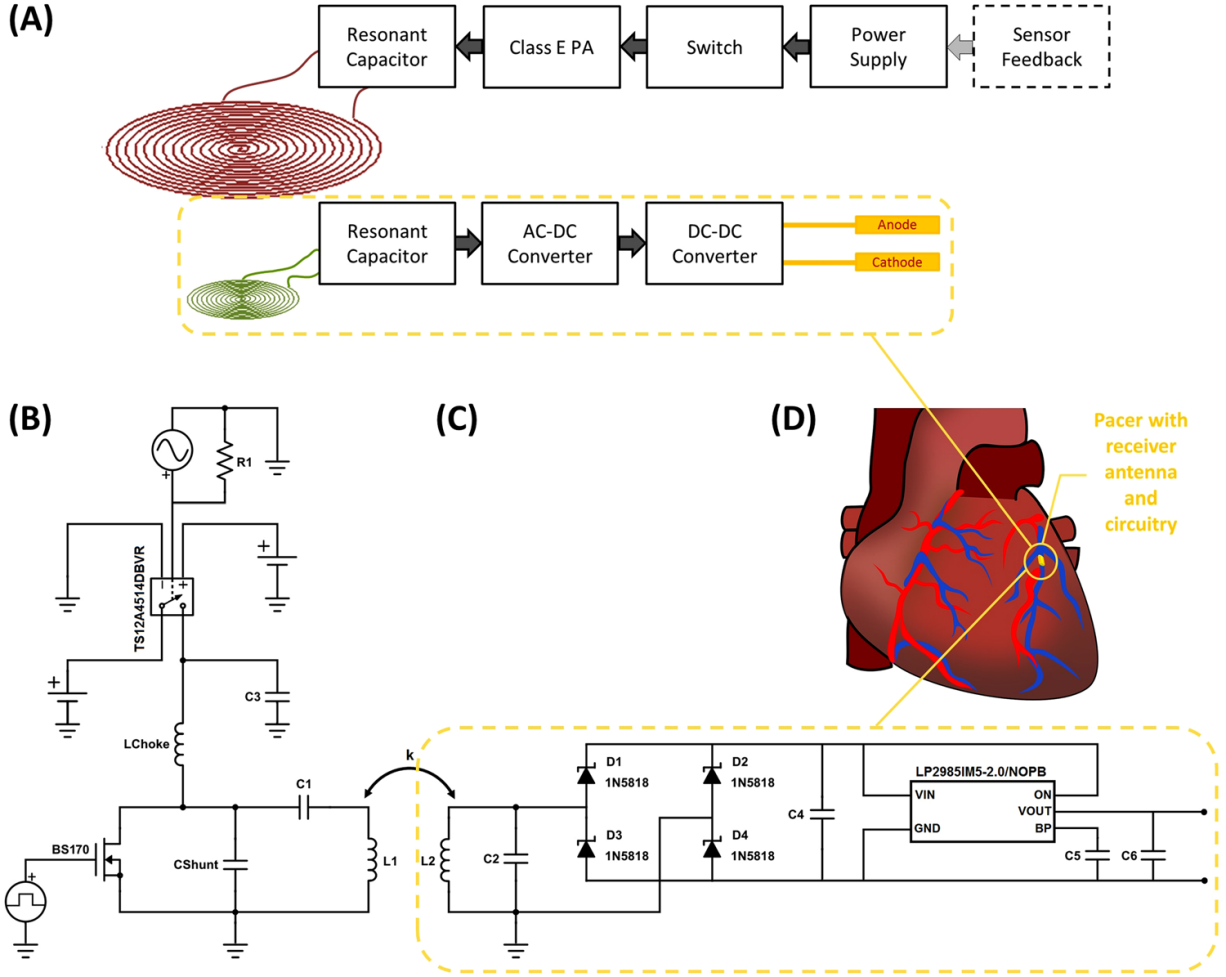
System Architecture. (**A**) A block diagram highlights the wireless pacing system with “remote-controlled” stimulation on the receiver side using intermittent power transfer from the transmitter side. (**B**) The transmitter circuitry consisted of the primary functional components of the pacemaker that remotely control and power the stimulator on the heart, including a switch, potential control input from a sensor, Class-E PA, and series resonant tank circuit. (**C**) The receiver circuitry consisted of only the components necessary to output a regulated voltage to the two electrodes, including a parallel resonant tank circuit, bridge rectifier, and voltage regulator. (**D**) A cartoon diagram depicts the pacer implant location into the anterior cardiac vein, which is made possible through miniaturization using a remote-controlled stimulation system.

The wireless pacing system consisted of two physically isolated components as shown in the block diagram (Fig. 1A) and circuit diagram (Fig. 1B and C). The cardiac component was miniaturized by maintaining only the core essentials for delivering a stimulatory pulse to the heart muscle. Therefore, it consisted of no internal control mechanism and simply functioned as a transformation unit, acquiring an AC input from the parallel resonant tank circuit to convert into a DC output for cardiac stimulation (Fig. 1C). In addition, this pacing module did not encompass any charge storage unit, such as a battery or capacitor. Its activity was entirely controlled remotely via intermittent power delivery from the transmitter at short pulses in the range of 0.1 to 1 ms in duration.

The entirety of the functional components was integrated into the power delivery module, consisting of the logic circuitry, class E power amplifier (PA), and series resonant tank circuit (Fig. 1B). This component would be implanted in the thoracic cavity to wirelessly deliver power to the cardiac pacer module. An analog switch permitted power delivery at the desired heart rate and pulse width for intermittent power transfer to the tank, and ultimately the receiver on the epicardium.

The advantages of separating the stimulator from the control system are four-fold. The first lies in the size reduction through the removal of complex logic circuitry. The second resides in the reduction in power requirements of the remote device. Wired transmission is far more power efficient than a wireless approach. Thus, by placing the majority of components inside the transmitting module with direct connection to a battery, the overall power requirements of the device are substantially reduced. The third advantage lies in a further reduction in power consumption via delivery of several short-pulse wireless transmissions as opposed to a single long transmission that continuously supplies the circuit or charges a small charge storage unit. This intermittent-type powering mechanism is achieved with the receiver designed only to function as a stimulator with greater than 99.9% inactivity and with less than 0.1% of the time during which power is needed for pacing. In contrast, inclusion of additional functions would require continuous power supply. Consequently, while 99.9% of the time the pacer would be in a “standby” mode (i.e. not delivering a stimulatory pulse), some power would continue to be needed for maintaining the remaining functions. Power reduction through intermittent transmission is analogous to the method implemented by Bluetooth Low Energy (BLE), in which cyclic intervals of “activity” and “sleep” allow for power consumption primarily in the “active” periods. In our pacing system, “sleep” intervals have zero power consumption and “active” intervals consume power exactly equivalent to that necessary to stimulate cardiac tissue without additional losses from a logic circuitry. The transmitting circuitry will, accordingly, become active only when the switch allows passage of current into the system, thus, significantly reducing power losses in the non-ideal inductive power transmission circuit. The fourth advantage is the consequential reduction in tissue absorption as a result of decreased time of transmission. This reduction allows for increased amplitude for each short duration pulse to increase power transfer while remaining below SAR safety limits.

### Antenna design

The antenna design was guided by geometric constraints for the implants and requirements for the range of power transfer. The antennas were developed as planar spiral coils to maintain flexibility and to minimize size along the z-direction. The boundary condition for the overall inductor size was preset based on implant position, thus allowing variability only in the inductance and effective resistance according to Eqs (1) and (2).

The coil inductances were impacted by the number of turns, wire radius, and wire spacing within the coil’s geometric boundaries as determined by using the Modified Wheeler formula by Mohan *et al*.^26^. The AC resistance of the coil was impacted similarly by these factors, as shown in Eq. (4)^22^:

4 $${R}_{AC}=\frac{l}{2\pi a\sigma \delta }\,,$$

where *l* is wire length, *a* is wire radius, *σ* is wire conductivity, and *δ* is skin depth defined by Eq. (5)^22^.

5 $$\delta =\frac{1}{\sqrt{\pi f{\mu }_{o}{\mu }_{r}\sigma }},$$

where *µ*_*o*_ is the permeability of air, and *µ*_*r*_ is the permeability of copper.

While inductance must be maximized to improve Q, the impacting variables negatively influence coil resistance as shown in Eqs (4) and (5): (1) an increase in the number of turns increases wire length, leading to ↑ *l* and ↑ *R*_*AC*_, (2) a decrease in wire radius reduces surface area of conduction, resulting in ↓ *a* and ↑ *R*_*AC*_, (3) an increase in frequency decreases skin depth of conduction, leading to ↑ *f*, ↓ *δ*, and ↑ *R*_*AC*_. Furthermore, a reduction in the wire-to-wire spacing increases eddy current dampening and wire resistance. Thus, these parameters dictated the variations in coil designs^22^.

**Figure 2:**
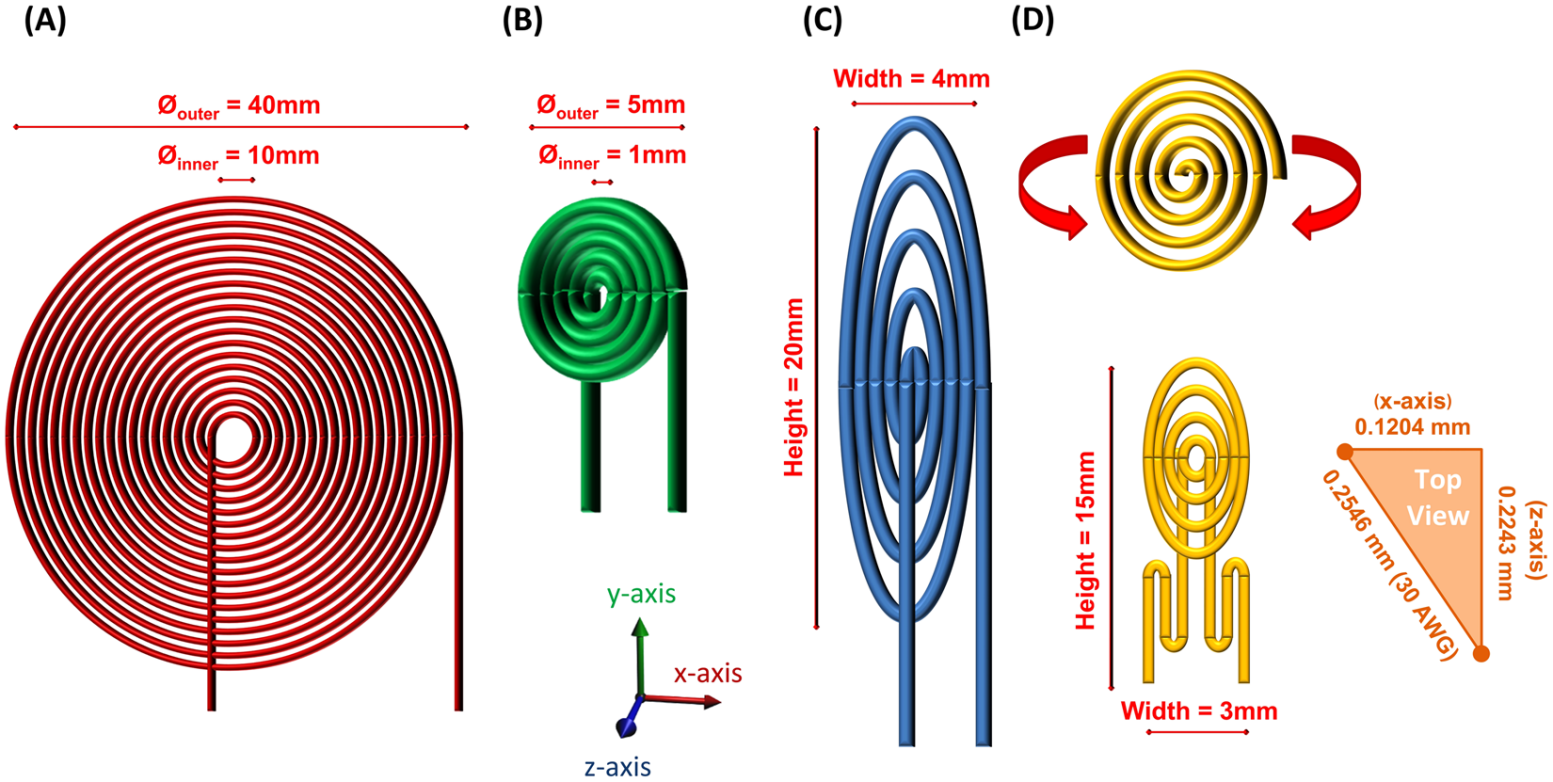
Antenna Design. (**A**) The transmitter coil was designed as a planar spiral coil with a 10 mm inner diameter, 40 mm outer diameter, 23 AWG wire, and 18 turns of the coil. (**B**) Receiver coil designed with 4 turns of 30 AWG wire with a circular geometry and no spacing between each turn resulting in a 5 mm outer diameter. (**C**) Receiver coil designed with 4 turns of 30 AWG wire with elliptical geometry without spacing between coils in the x-direction, resulting in a width of 4 mm, and over 2 mm of spacing between the coils in the y-direction, leading to a height of 20 mm, and (**D**) Receiver coil designed with 4 circular turns and 3 meandering turns of a 30 AWG wire. The upper figure highlights a coil with a circular geometry and spacing between coils equal to that of the wire diameter. This configuration wraps into the z-direction along the y-axis of the coil and forms into a half-cylindrical shape. This design was further supplemented by a meandering structure, resulting in a final antenna shape shown at the bottom-left with width of 3 mm, height of 15 mm, and depth of 2 mm. The lower right diagram demonstrates > 50% space reduction along the x-direction through the transfer of coil spacing into the diagonal.

The transmitter, to be implanted into the thoracic cavity anterior to the cardiac chambers, is permitted adequate space. A 40 mm diameter planar spiral coil was fabricated with a 23 AWG copper wire, spaced apart by a distance equal to that of the wire diameter with 18 turns of the coil (Fig. 2A).

**Figure 3:**
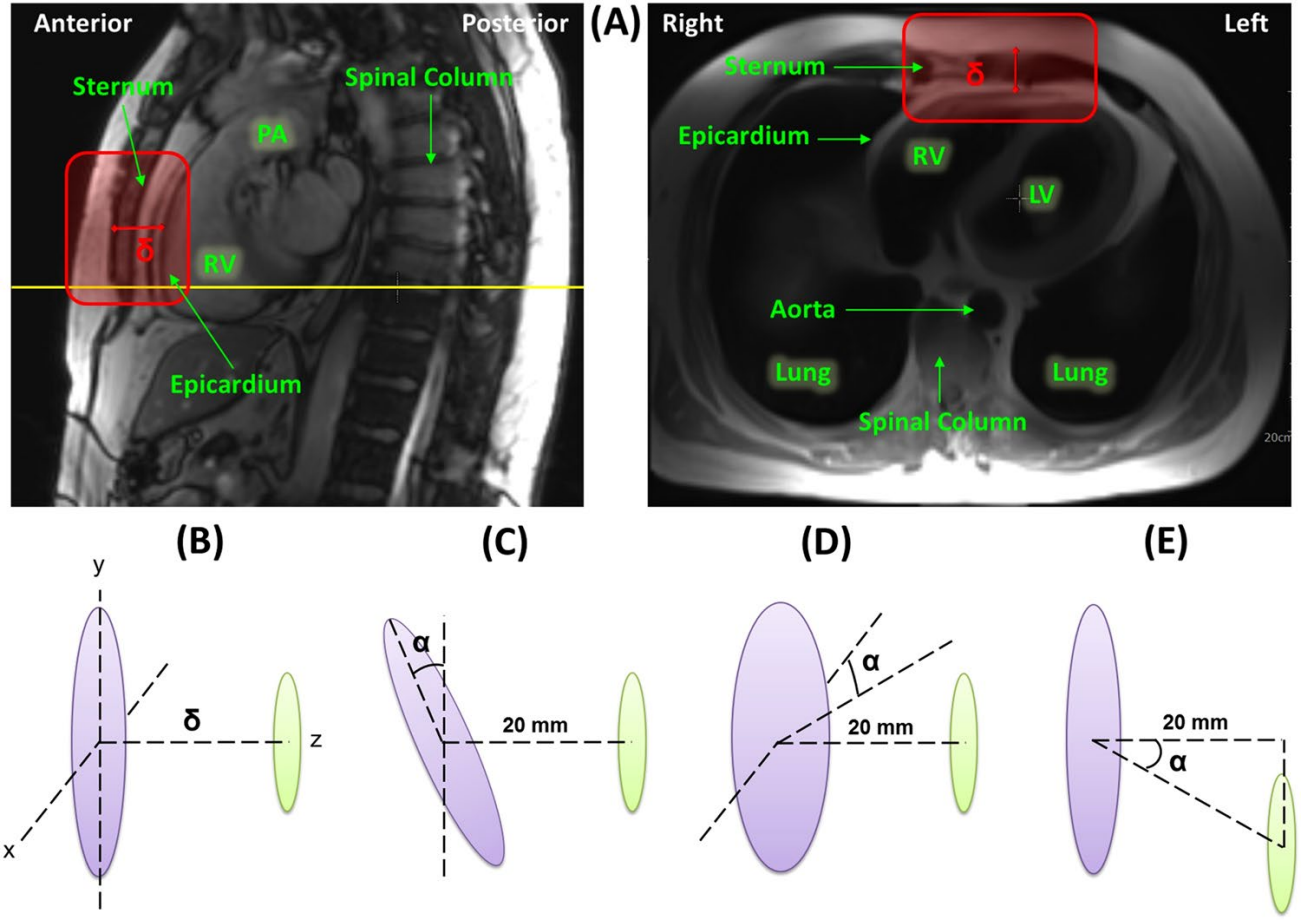
Determining Range of Function. (**A**) Thoracic MR images in which the left image shows the sagittal view with the yellow line representing the cross-reference line for the axial black blood image in right image. The red box represents the region in which measurements were made in both views. The distance, δ, represents the displacement measured along the region of the red box in which the receiver will be located in the anterior cardiac vein and the transmitter will be located in the anterior chest wall below the adipose tissue along the sternal border. PA: pulmonary artery, RV: right ventricle, LA: left ventricle. (**B**) Antenna assessment at distance, δ, (**C**) Antenna assessment with x-axis angular misalignment at angle, α, (**D**) Antenna assessment with y-axis angular misalignment at angle, α, and (**E**) Antenna assessment with displacement misalignment at angle, α.

The cardiac pacing unit contains the receiver antenna and is aimed to be implanted inside the anterior cardiac vein (diameter of 3.8 ± 0.7 mm)^27^. This location provides a compromise in minimizing distance to the transmitter, while maximizing the space for a larger implant and maintaining ease-of-access for catheter-based deployment. Three different coil designs were investigated with 4 turns of a 30 AWG wire: (1) a coil with a circular geometry and no spacing between each turn (Fig. 2B), (2) a coil with an elliptical geometry with no spacing between coils in the x-direction and over 2 mm of spacing between coils in the y-direction (Fig. 2C), and (3) a coil with a circular geometry and spacing between coils equal to that of the wire diameter that forms into a half-cylindrical shape via a gradual fold in the z-direction along the y-axis of the coil; this configuration was further supplemented by a meandering trace with three turns of the coil (Fig. 2D). The wrapped modification into a half-cylinder achieves a smaller size in the x-direction, while maintaining distance between the turns of the coil along the diagonal direction to minimize proximity effects and capacitive coupling. Furthermore, the addition of the meandering structure to the spiral-shaped antenna can improve power transfer efficiency over a greater range despite misalignment. The meander allows for the placement of a condensed linear antenna structure with an alternative field pattern in a small space to enhance power absorption. Thus, the antenna provides sufficient power transfer in the setting of dynamic cardiac contractions, which create variations in the transmitter-to-receiver transmission angle^28^.

### Circuit design

The system was designed using a carrier frequency of 13.56 MHz based on frequency band assignments for medical devices and tissue absorption criteria in consideration with power transfer efficiency goals^29–31^. The resonant capacitor, *C*_*res*_, for each corresponding inductor was computed using Eq. (6)^22^:

6 $${C}_{res}=\frac{1}{L{(2\pi f)}^{2}}$$

Power transfer for an inductive link is maximized when LC tank circuits of the transmitter and receiver are both tuned to the same resonant frequency, in this case 13.56 MHz (Table 1).

**Table 1:** Summary of theoretical computations.

Summary of Theoretical Computations				
Parameter	Transmitter	Circular Receiver	Elliptical Receiver	Cylindrical Receiver
L (nH)	9940	160	225	134
C (pF)	14	890	610	1040

A Class E PA was implemented for the transmitter for its capacity to maintain high efficiency with low DC input current and high AC output current into the tank circuit. The Class E PA was designed based on principles described by Sokal^42^. In the selection of the MOSFET, essential parameters included the low on-resistance (R_DS(ON)_), low output capacitance (C_oss_), and low gate threshold voltage (V_GS(th)_).

## Experimental Design

### Comparison of power transfer efficiency

The range for power transmission was assessed via thoracic Magnetic Resonance Images (MRI) to determine the anatomic displacement between the transmitting and receiving modules. All imaging studies were approved by the UCLA Institutional Review Boards (IRBs) and informed consent was obtained from participants. Measurements were made at six equidistant points along the red box highlighted in Fig. 3A and the average value was used to estimate the displacement between the right ventricular (RV) free wall, near the septal wall and the RV apex, and the anterior chest wall, below the adipose tissue, at the sternal border. Adipose tissue anterior to the sternum poses the greatest source of variation in the distance between the transmitter and receiver. For this reason, in bypassing the fat layer, we can better predict the interaction between the transmitter-receiver pair and improve power transfer efficiency.

**Figure 4:**
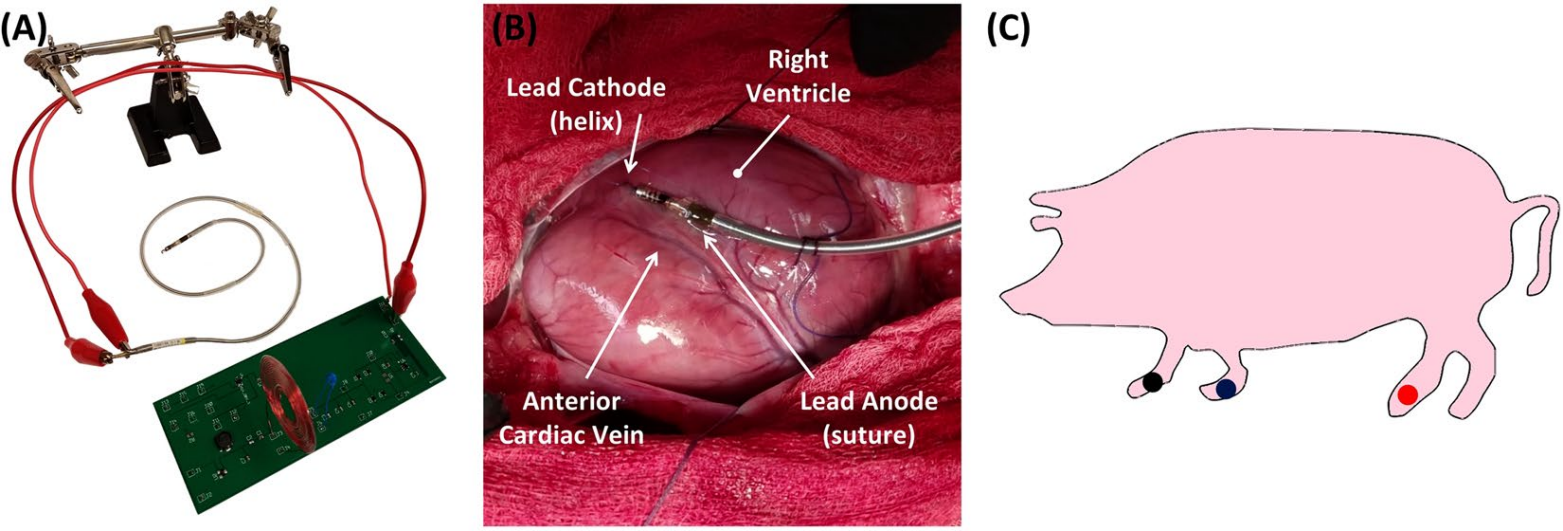
Experimental Setup. (**A**) PCB with transmitter and receiver circuitry and the output of the receiver electrically connected to the anode and cathode of an existing bipolar lead; (**B**) implant location of the bipolar lead on the porcine heart with the cathode at the distal tip fixated via helix into the epicardial wall and the anode proximal to the cathode in the form of a ring fixated via sutures; (**C**) ECG lead placement shown via the blue, red, and black dots.

The transmitter and receiver circuitry were printed on a Printed Circuit Board (PCB) (Fig. 4A) and the antennas soldered into the circuit. The power transfer efficiency with each of the three types of receiver antennas in Fig. 2B–D were tested under four conditions as shown in Fig. 3B–E: (1) distance between the transmitter and receiver antennas from 20 to 40 mm apart, (2) x-axis angular misalignment between antennas up to 45 degrees with a distance of 20 mm apart, (3) y-axis angular misalignment between antennas up to 45 degrees with a distance of 20 mm apart, and (4) displacement misalignment between antennas up to 45 degrees with a distance of 20 mm apart.

Bench tests were performed using a 500 ohm load, based on expected impedance from the electrode, tissue-electrode interaction, and tissue in the porcine heart experiments.

### A euthanized porcine model to demonstrate *ex vivo* pacemaker functionality

A postmortem study was performed to demonstrate the feasibility of our pacing system in a male Yucatan miniature pig (S & S Farms, Ranchita, Calif). All animal studies were approved by the UCLA Office of Animal Research in compliance with the UCLA IACUC protocols. A thoracotomy was performed to expose the epicardium for external pacing by an experienced veterinarian from the UCLA Department of Animal and Laboratory Medicine. The transmitter and receiver antennas were aligned and spaced at 20 mm apart. The output of the system was connected to electrodes of an existing standard impedance bipolar lead (Fig. 4A), the St. Jude Tendril SDX Model 1388 T with previously reported short-term ventricular lead impedance of 553 ± 106 ohms^32^. The lead was fixated into the epicardium of the pig heart near the anterior cardiac vein at the RV apex. The distal electrode, acting as the cathode, was electrically connected to the fixated helix; the proximal electrode, acting as the anode, was sutured in place (Fig. 4B).

External pacing was initiated immediately post-euthanasia to minimize cellular apoptosis and release of intracellular electrolytes (Na^+^, K^+^, Ca^2+^). A pacing amplitude of 2 V, pulse width of 1 ms, and heart rate of 60 BPM were delivered via the bipolar lead electrodes. Simultaneous Electrocardiogram (ECG) recordings allowed for monitoring cardiac rhythm and assessing pacemaker effectiveness (Fig. 4C).

Data availability: The data generated during and/or analyzed during the current study are available from the corresponding author on reasonable request.

## Results

### Comparison of power transfer efficiency

**Figure 5:**
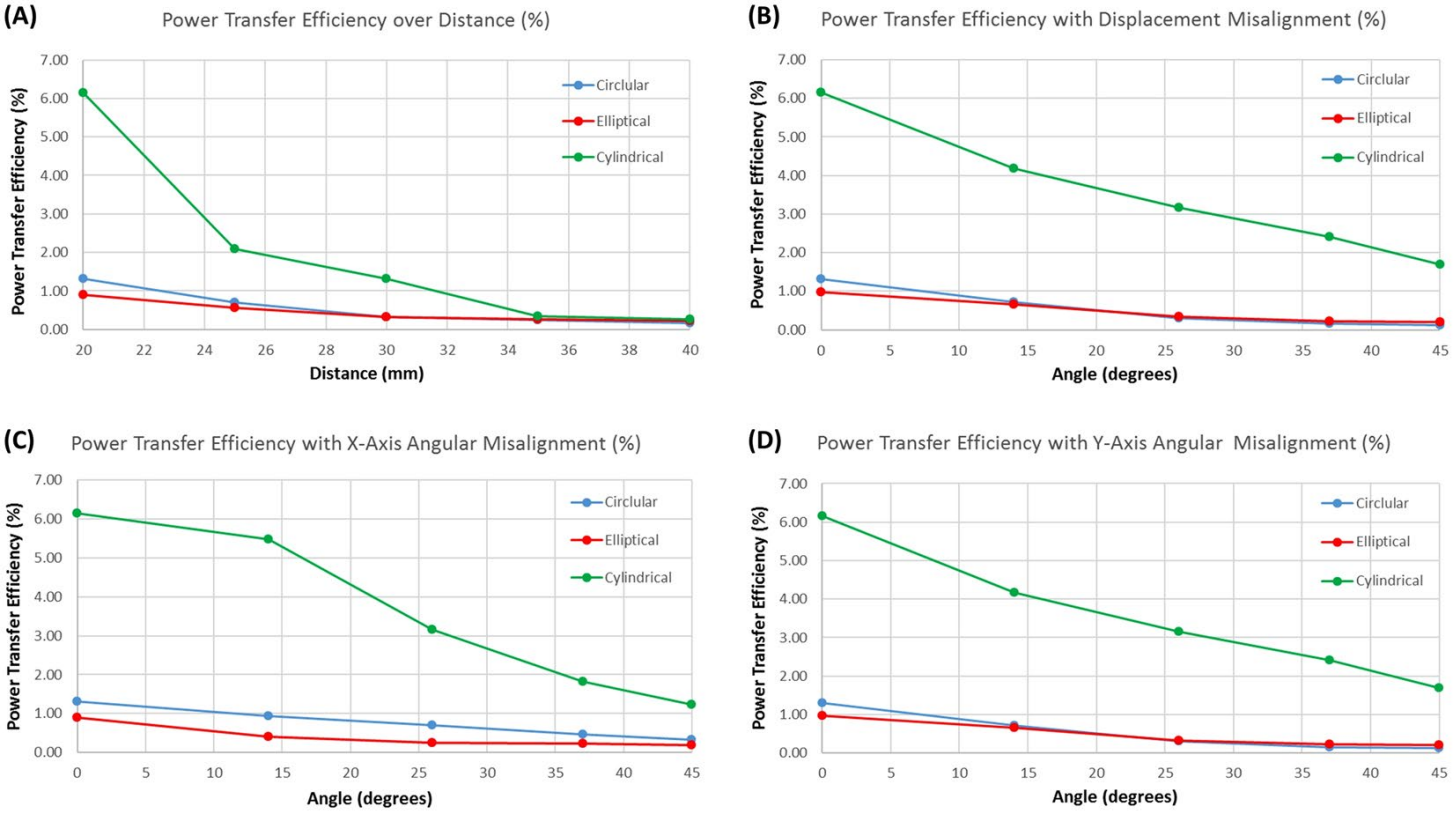
Power Transfer Efficiency Measurements. (**A**) A range of distances from 20 mm to 40 mm at intervals of 5 mm. (**B**) Displacement misalignment from 0 to 45 degrees. (**C**) X-axis angular misalignment from 0 to 45 degree. (**D**) Y-axis angular misalignment from 0 to 45 degree. The wrapped half-cylindrical antenna with the meandering structure provided the optimal power transfer efficiency as compared to the circular and elliptical configurations.

MRI displacement measurements revealed an estimated transmitter-to-receiver distance of 2.0 ± 0.85 cm, which is within the range of the testing criteria of Fig. 3B–D. We compared the power transfer efficiency (%) among the three different receiver antenna designs: 1) a coil with circular geometry without spacing between each turn, 2) a coil with elliptical geometry without spacing between coils in the x-direction, and 3) a coil with a circular geometry and spacing equal to that of the wire diameter with the coil wrapped into a half-cylindrical shape with a meandering tail structure (Fig. 2D). While the power transfer efficiency invariably diminished in response to an increase in distance (Fig. 5A), displacement angular misalignment (Fig. 5B), x-axis angular misalignment (Fig. 5C), and y-axis misalignment (Fig. 5D) between the transmitter and receiver antennas, in all cases, the data indicated that the wrapped half-cylindrical antenna provided the optimal power transfer efficiency.

Given a transmitter supply of 5 V at 0.20 A, the half-cylindrical receiver antenna was capable of reaching the 2 V voltage amplitude with a 500 ohm load at >3 cm without misalignment, 2 cm with 45° displacement misalignment, 2 cm with 45° x-axis angular misalignment, or 2 cm with 45° y-axis angular misalignment. Despite continuous wireless transmission requiring 1 W of power, intermittent power transmission at 1 Hz and 1 ms pulse width engendered a power reduction to 1 mW. Also notable is that testing was performed with a 1 ms pulse width, as opposed to the commonly performed pulse width of < 0.5 ms, which further reduces the power requirement.

### Porcine model to demonstrate pacemaker functionality

**Figure 6:**
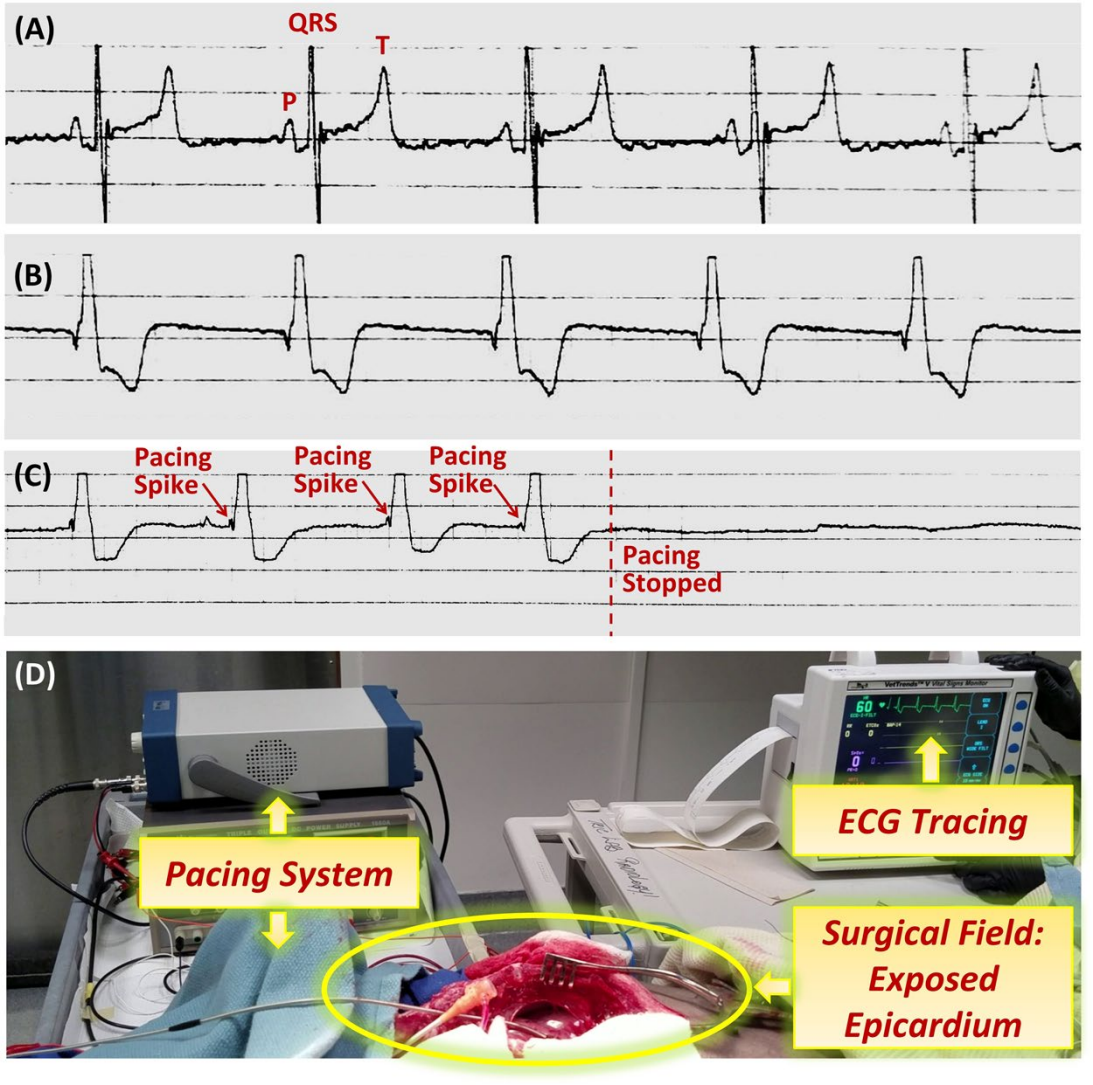
Animal Experiments. (**A**) Prior to euthanasia of the animal, ECG recordings revealed a heart rate of 77 BPM, MAP of 90 mmHg, and normal P, QRS, and T waves that indicate normal sinus rhythm. (**B**) After euthanasia and during the pacing period, ECG recordings revealed a heart rate of 60 BPM and MAP of 37 mmHg. The wide-complex paced rhythm indicates successful pacing via the remote controlled wireless pacing system. (**C**) Zoomed-in view of the ECG tracing highlights the pacing spikes that appropriately stimulates ventricular contraction. When pacing was deactivated, electrical activity was absent as indicated by a flat line on the ECG, thus supporting the pacer-dependency of the post-mortem animal’s heart and effectiveness of the remote-controlled pacemaker’s ability to stimulate appropriate electrical activity in the mammalian heart. (**D**) Wide-angle image highlights the setup for post-euthanasia pacing of a pig heart accompanied by simultaneous ECG recording.

Based on bench test data of transfer efficiency, the wrapped half-cylindrical receiver antenna with the meandering structure (Fig. 2D) was determined as the optimal design for the *ex vivo* investigation of the pacemaker. To determine the efficacy of paced cardiac rhythms, we assessed the surface ECG readings pre-euthanasia, post-euthanasia, and during experimental pacing. Prior to euthanasia, the ECG exhibited a typical P wave for atrial contraction, QRS for ventricular depolarization, and T wave for ventricular repolarization (Fig. 6A). Post-euthanasia, epicardial stimulation generated a wide-complex paced rhythm as anticipated for ventricular pacing (Fig. 6B). During the pacing period, we were able to restore mean arterial blood pressure (MAP) from 0 to 37 mmHg. Thus, our *ex vivo* pacing via the wrapped half-cylindrical antenna and remote stimulation control provided sufficient power to energize a postmortem swine heart (see Supplementary Video 1).

## Discussion

The novelty of this work lies in a remote-controlled inductive power transfer system design to mitigate lead-related complications. Our strategy for a leadless pacemaker lies in the unique architecture that positions the logic circuitry within the transmitting component, such that the pacing module is miniaturized and power requirements are greatly reduced by intermittent power transmission. Size reduction holds promise to enable pacer implantation in the anterior cardiac vein (diameter of 3.8 ± 0.7 mm) to eliminate mechanical failure points of existing implantable devices. We demonstrated that a wrapped half-cylindrical antenna design with a meandering tail structure provides optimal power transfer efficiency within these geometric constraints. *Ex vivo* studies in the Yucatan miniature pig further substantiated the feasibility of this remote-controlled pacing system by achieving a paced rhythm at 60 BPM while requiring as low as 1 mW for power transmission.

Our pacing system is proven more effective than currently marketed leadless devices through the exclusion of a pacer-integrated battery. These batteries increase the size and weight of the device, leading to repetitive mechanical burden on the fixation system that is inserted into the ventricular myocardium to anchor the pacer (nitinol tines for Micra and non-retractable helix for Nanostim). These suboptimal fixation systems have introduced risks for RV perforation with tamponade in addition to excessive bleeding and possibility of device embolization during implant^9^. Furthermore, battery life becomes a limiting factor as extraction of an expired device can be hazardous due to rapid fibrotic encapsulation in the time post-implant. As a result, in the cases of battery exhaustion or device failure, rather than replacement, additional pacers may be implanted in the limited real estate of the RV chamber, thus restricting device implantation primarily to much older patients^10^.

Inductive power transfer systems have been extensively evaluated as a means to power medical implants. The placement of the control system in the receiver unit, however, has often hindered progress due to the need to provide continuous power to the logic circuitry despite only requiring intermittent power delivery for stimulation. To accommodate a continuous power drain, a rechargeable battery is often integrated into the receiver unit to supply the system; its charge is then periodically restored via inductive power transfer to a receiving coil^16–18^. This mechanism, however, results in increased size of the cardiac component.

Inductively powered devices without integrated batteries have been proposed with the ability to deliver sufficient power for the circuitry, but nevertheless insufficient power for stimulation in the absence of some charge storage unit. Continuous midfield powering has been utilized to power a cardiac implant at a 5 cm range by coupling 500 mW into tissue and achieving nearly 200 µW while remaining below SAR safety thresholds^19, 20^. The power requirement of a pacemaker chip has been reported as low as 8 µW^33^, thus significantly below the achieved power transfer. The main issue, however, arises from stimulation pulses. Within one week post-implant, standard impedance lead electrodes reach about 500 ohms and high impedance lead electrodes reach about 1000 ohms^34^. While pacing amplitudes may range from 0 to 5 V, the average pacing amplitude is about 0.8 V^35^. Given this value, direct wireless transmission via midfield is insufficient to generate a stimulatory pulse with 500 mW coupled into the tissue. While midfield promises about 2 mW of power transfer at the maximum safety threshold of SAR limits^20^, this requires higher energy coupled into the system, which may be impractical for a pacemaker.

Given 1 mW of power, our current system is capable of achieving a voltage amplitude of 2 V with 500 ohm impedance at the displacement range estimated from MRI measurements with up to 45° misalignment in all directions (Fig. 3). While our current system achieves more than 2-fold the average pacing amplitudes, we anticipate achieving a voltage amplitude of up to 5 V in the final device to match the thresholds of existing devices. The increased safety margin will allow us to accommodate a larger patient population. This can be made possible by increasing the input power, which is still well below SAR limits. Various design adjustments can also be made to improve efficiency.

Further improvements can especially be made through enhancements in the antenna design. While the planar geometry of the transmitter antenna allows for flexibility, it substantially reduces inductance and power transfer efficiency. A two-layer and more closely packed design may improve efficiency while maintaining versatility. The miniature pacer receiver antenna design can also be modified to improve adaptability with changes in the alignment angle. The receiver antenna can especially benefit from migration into a flexible PCB to allow for complex and multi-layered antenna designs while remaining within the constraints of the anterior cardiac vein. This can greatly enhance antenna Q and wireless power transfer range.

The current pacer circuitry has two primary sources of power loss: (1) bridge rectifier diodes and (2) voltage regulator. Each component results in a nearly 300 mV drop, thus reducing voltage by a total of nearly 1 V prior to output. A MOSFET-based approach for AC-DC conversion, such as the LT4320 ideal diode bridge controller^36^, may improve efficiency while remaining within the size limits of the anterior cardiac vein. Changes in the input and output capacitors of the regulator can also help reduced output ripple. A switching regulator may alternatively be used instead of a linear regulator to avoid heat production.

The presented work demonstrated pacing by connecting the output of the receiver circuit to an existing bipolar pacing lead. Future work will consist of two electrodes electrically connected to the receiver circuit in a single miniature leadless packaging to be inserted into the coronary veins via catheter delivery. Furthermore, our experiments were performed via a standard impedance lead. Many studies have been performed on high impedance leads, which result in a decrease in the voltage amplitude for pacing^34^. High impedance electrodes with improved contact for induction of cardiomyocyte depolarization can be developed to decrease power requirements further and to improve range of function.

Lastly, pacemakers are often a combination of a sensor and stimulator, in which the sensor defines intervals during which the pacer enters an “active” mode when the patient’s heart cannot maintain a heart rate above a programmed threshold and exits into a very low-power “sleep” mode at all other times. The current system remains in an active stimulation mode at all times to maintain a continuous 60 BPM pulse. Future designs will include feedback information from a sensor inside the transmitter unit to initiate a series of pulses in response to detecting arrhythmia. This will further reduce power consumption as the pacing circuit will only activate when the patient’s own heart cannot sustain a normal rhythm. Security measures will also be necessarily implemented for the ultimate clinical application of the pacemaker. This may include the use of miniature fuse structures, tuning offsets, or encryption techniques as a means to maintain patient safety under malicious attacks^37–41^. This combination of a secure remote miniaturized stimulator with sensor feedback can ultimately provide increased patient safety for the next generation of implantable devices.

## Electronic supplementary material

Supplementary Video

Supplementary Information
