# Supplementary material for: Inductively powered wireless pacing via a miniature pacemaker and remote stimulation control system
Source: Sci Rep. 2017 Jul 21;7:6180. doi: 10.1038/s41598-017-06493-5 (PMC5522478; doi:10.1038/s41598-017-06493-5)

Supplementary Information:

Inductively Powered Wireless Pacing via a Miniature Pacemaker and Remote Stimulation Control System

Parinaz Abiri [a,b], Ahmad Abiri [b], René R. Sevag Packard [a], Yichen Ding [a,b], Alireza Yousefi [c], Jianguo Ma [a,1], Malcolm Bersohn [d], Kim-Lien Nguyen [d], Dejan Markovic [c], Shervin Moloudi [c], Tzung K. Hsiai [a,b*]

[a] Department of Medicine, David Geffen School of Medicine at UCLA, Los Angeles, CA 90095, USA

[b] Department of Bioengineering, School of Engineering and Applied Sciences, UCLA, Los Angeles, CA 90095, USA

[c] Department of Electrical Engineering, School of Engineering and Applied Sciences, UCLA, Los Angeles, CA 90095, USA

[d] VA Greater Los Angeles Healthcare System, 11301 Wilshire Blvd, Los Angeles, CA 90073, USA

*Corresponding Author: THsiai@mednet.ucla.edu

This file includes:

Video 1: Video recording of cardiac pacing post-euthanasia. Pacing via the remote-controlled wireless pacemaker results in cardiac contractions. When pacing is deactivated, contraction ceases

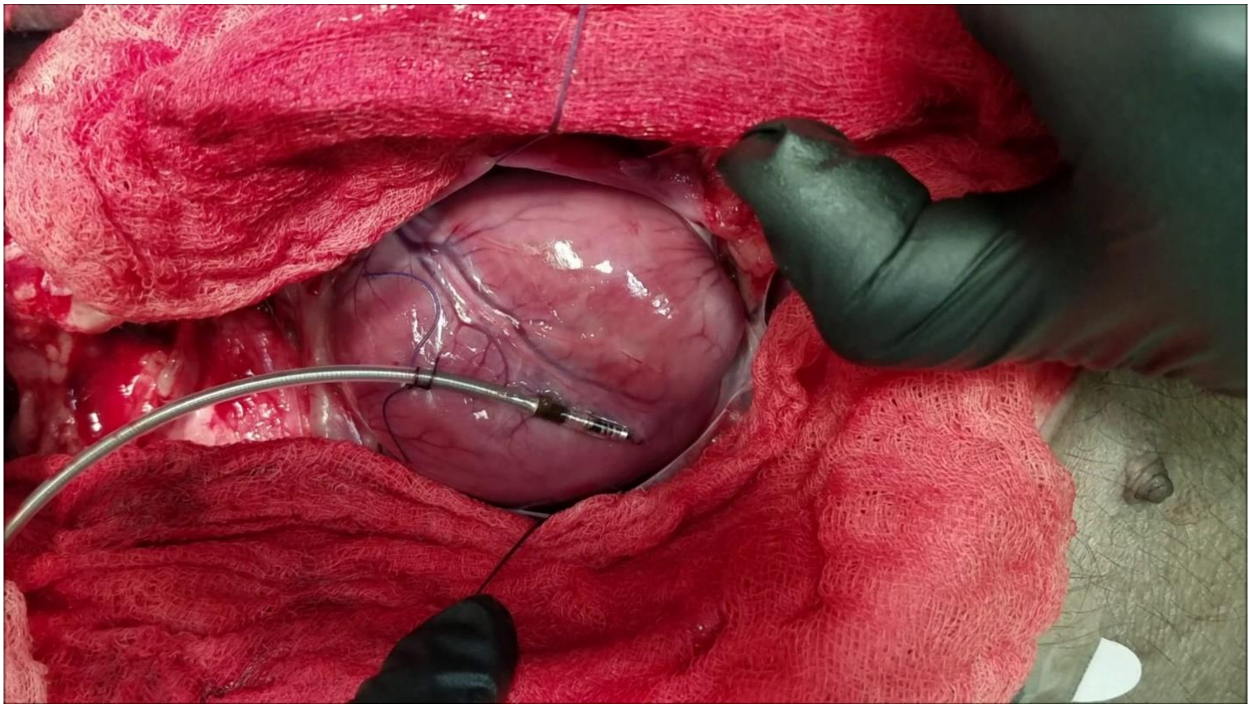

Supplement: Supplementary file 2 — Supplementary Information [file 41598_2017_6493_MOESM2_ESM.pdf]
